# Bread from Stones

**Published:** 1894-11

**Authors:** 


**Bread from Stones.** A new and rational system of Land Fertilization and Physical Regeneration. Translated from the German. Philadelphia, Pa., A. J. Tafel, 1011 Arch Street, 1894. Price, 25 cents.

This little book is a protest against the universal custom by the civilized world of fertilizing fields and gardens with stable manure, Chili nitre, and other artificial compounds. In its place the author, Dr. Hensel, proposes the use of stone meal. This consists of an extremely fine powder produced by the careful grinding of rocks of different composition intelligently mixed together, according to the constituents that each sample of rock contains.

He attributes the various diseases that afflict fruits, vegetables, and grains to the foul messes served to them for food under the name of fertilizers. He intimates that the unhealthiness of milk may be attributed to the same cause, and declares that all these diseases of plants may be abolished by the use of stone meal.

He would abolish all other sources of the enriching of the ground for agricultural purposes. Human ingenuity has been exerted to its utmost to find new sources for fertilization. The Gauno Islands are well known as a mine of wealth to the State of Peru for their vast beds of bird-lime which is exported all over the world for improving soils. The so-called Chili nitre beds, which really contain nitrate of lime, are another example.

England avails herself of her hold upon Egypt by digging up the bodies of the ancient inhabitants which have remained in the ground for two thousand years and more, transporting them to the British Isles, running them through a bone-mill, and then scattering the dust over the soil to increase its fertility.

Competent Egyptologists have estimated that there are not less than six hundred millions of mummified bodies buried in the earth of Egypt. It gives one rather a curious sensation to reflect that the ancient Egyptians should have taken such pains to preserve the bodies of their ancestors only to save them for an English bone-mill.

In America, pebble phosphates are found in Florida and similarly used for fertilizing. These beds are noticed in the October number of *The Engineering Magazine* at page 155.

Finally, William B. Phillips, Ph. D., has contributed to *The American Institute of Mining Engineers*, a paper advocating the use of phosphate slag as a fertilizer. This slag is produced by the use of lime in the Bessemer converter in the manufacture of steel. The lime extracts the phosphorus from the molten metal and so improves the quality of the steel. The resulting slag is then ground up and sold as a fertilizer.

(V. *Proceed. Am. Inst. Min. Eng.*, vol. XVII, p. 84.)

All these varied means of obtaining fertilizers are rejected by Dr. Hensel, and "nature's manure," the impalpable dust of the rocks, is to be substituted.

This method of fertilizing is not a mere theory but has been actually tried with success, and letters from farmers who have tried it and who approve of it appended.

